# Overweight, obesity and related conditions: a cross-sectional study of adult inpatients at a Norwegian Hospital

**DOI:** 10.1186/1756-0500-7-115

**Published:** 2014-02-27

**Authors:** Ingrid Sørdal Følling, Bård Kulseng, Anne-Sofie Helvik

**Affiliations:** 1 Department of Health Sciences, North-Trøndelag University College, Røstad, 7600 Levanger, Norway; 2 Department of Public Health and General Practice, Faculty of Medicine, Norwegian University of Science and Technology, Postboks 8905 MTFS, 7491 Trondheim, Norway; 3 Research Centre for Overweight and Obesity, St. Olavs University Hospital, Olav Kyrres gt. 6, 7006 Trondheim, Norway; 4 Department of Cancer Research and Molecular Medicine, Norwegian University of Science and Technology, Postboks 8905, N-7491 Trondheim, Norway; 5 St. Olavs University Hospital, Trondheim, Norway

**Keywords:** BMI, Overweight, Obesity, Epidemiology, Hospital

## Abstract

**Background:**

Overweight, obesity and associated conditions are major public health concerns in Norway. The prevalence of overweight and obesity in the general population in Norway is increasing, but there are limited data on how the situation is in hospitals. This study aimed to find the prevalence of overweight and obesity, and explore the associations of overweight, obesity and its related medical conditions in an adult in-patient sample at specified somatic and psychiatric departments at St. Olavs Hospital, Trondheim.

**Results:**

A total of 497 patients participated. The mean BMI for the total sample at screening was 25.4 kg/m^2^. The prevalence of overweight and obesity was 45.1%. There was a higher association of overweight and obesity among patients aged 40–59 years (OR: 1.7) compared to those being younger. There was no significant difference between the somatic and the psychiatric samples. In the somatic sample overweight and obesity was associated with obesity-related conditions for both genders (OR: 2.0 and 2.1, respectively), when adjusted for age.

**Conclusion:**

The substantial prevalence of overweight and obese patients may pose a threat to future hospital services. To further address the burden of overweight and obesity in hospitals, we need more knowledge about consequences of length of stay, use of resources and overall cost.

## Background

During the past decades it has been reported an increasing prevalence of overweight (BMI ≥ 25 kg/m2) and obesity (BMI ≥30 kg/m2) worldwide [1,2]. Also in Norway, the prevalence of overweight and obesity has increased over the last twenty years [3]. Type 2 diabetes mellitus (T2DM), cardiovascular diseases (CVD) and cancer are by far the leading cause of mortality in the world, and they are all associated with overweight and obesity [4]. There is an increased disease risk in adults with excess abdominal fat and high body mass index (BMI) [5]. The associated socio-economic costs are extremely high and also likely to increase [6].

In clinics and public hospitals in the United States (USA) studies of overweight and obesity among adult outpatients has shown prevalence rates around 80% [7,8]. It is found that obesity itself significantly extend the length of stay in hospital of patients treated for different causes [9]. Further, among hospitalized patients 75.0% had one or more obesity-related condition(s) [7]. A Brazilian study of admissions at hospitals showed that diseases associated with overweight and obesity had a significant impact on hospitalizations [10]. In Ireland, hospital discharges related to overweight and obesity has increased alarmingly [11]. A Swedish cohort study found that overweight and obesity implied increased risk for hospitalization [12].

In Norway there are no studies targeting the prevalence of overweight, obesity and the associated conditions in hospital settings. Thus, the aim of this study was to find the prevalence of overweight, obesity and explore the associations of overweight, obesity and its related medical conditions in an adult Norwegian hospitalized sample.

This article reports an image of the impact of overweight, obesity and its related diagnoses at a Norwegian hospital.

## Methods

This study was based on a cross-sectional study design where adult inpatients at selected departments at St. Olav’s Hospital, Trondheim University Hospital were assessed at a specified time point.

### Participants

The study sample consisted of 497 adult patients (18 years or older) from somatic (n = 356) and psychiatric (n = 141) departments, who were hospitalized a specific day and hour in spring 2007. Totally 532 patients were hospitalized at selected departments at the inclusion time, but 23 patients were excluded from measures (terminal or too somatically ill = 11, to disabled = 4, in a too bad psychiatric shape = 7, pregnant women = 1) and 12 patients declined to participate (93.4% participation rate).

### Measures

Gender, age, height and weight (for BMI), and diagnoses were collected. Standardized height measurement was used to measure height. Height was measured without shoes and noted to the nearest centimeter. Newly recalibrated hospital scales were used to do the measure of weight. The subjects were weighted wearing light clothing and without jacket, shoes or outdoor garments, and the weight were noted to the nearest half kilogram. BMI was calculated and criteria for underweight, normal weight, overweight, and obesity used in the present study were consistent with the definitions set forth by the World Health Organization (WHO) where underweight = BMI ≤18.5 kg/m^2^, normal weight = BMI 18.5-24.9 kg/m^2^ overweight = BMI 25.0-29.9 kg/m^2^ and obesity = BMI ≥30 kg/m^2^[13]. Obesity-related conditions used were those diagnoses with well-established epidemiological associations with obesity: T2DM (E11-E 14), hypertension (I10- I 15), dyslipidemia (E 78.0-E 78.9), and coronary artery disease (I 20- I 25, I 70- I 79), osteoarthritis (M 15- M 19), cerebral vascular accident (I 60- I 69), cholelithiasis (K 80) and sleeping apnea (G 47 3) [14].

### Study procedures

Patients who gave informed consent to participate, or had measured data from the hospital records to do a retrospective chart review, were included in the data collection. The data was collected by trained nurses at each selected department at the hospital. The patients who did not have their height and weight in their chart, got an information letter where they were informed about the nature of the study, and they reported back to the responsible nurses their personal consent. All data collected were anonymous; there were no names of individuals, or other id number referring back to the participants. The survey was approved by the Norwegian Data Inspectorate and the Regional Ethical Committee for Medical Research.

### Statistical analysis

Statistical Package for the Social Sciences for Windows (SPSS Inc. Illinois, US, version 16.0) was used to analyze the data. The material was controlled before the analysis.

The continuous variables for males and females were tested for normality by results of the Kolmogorov-Smirnov Statistic. This was done separately for males and females. All the continuous variables had significant results (p < 0.0001), suggesting violation of the assumption of normality. They were transformed to fit the normality distribution and independent sample t-test with and without equal variance (based on Levene’s test) was further used to investigate difference in gender, and difference at somatic and psychiatric departments for each of the continuous variables.

Logistic regression analysis (with the method Enter) was first used to study associated factors with having a BMI tied to overweight and obesity (≥ 25 kg/m^2^) vs. underweight and normal weight (≤ 25 kg/m^2^) together. Unadjusted and adjusted logistic regression analyses, were performed by departments (somatic and psychiatric), gender and age strata as independent variables. The second outcome, in the somatic sample, having obesity-related conditions or not, was studied in crude and adjusted odds ratio analysis stratified by gender. BMI categories and age strata were independent variables that were adjusted for. Gender and age had an interaction effect. Statistical significance was set at p ≤ 0.05 for all comparisons.

## Results

### Sample characteristics

A total of 497 hospitalized adult patients at St. Olavs Hospital participated in the study, of whom 243 (48.8%) were males. The mean BMI for the total sample at screening was 25.4 kg/m^2^. The general characteristics (age, height, weight and BMI) of the male and female patients studied according to somatic and psychiatric departments are presented in Table 1.

**Table 1:** Basic characteristics of study sample by departments and gender (N = 497)

	***Somatic (N = 356)***		***Psychiatric (N = 141)***	
***Variables***	**Males (n = 194)**	**Females (n = 162)**	**Males (n = 49)**	**Females (n = 92)**
**Age(yrs)**	65.27 (17.75)	63.20 (19.91)	38.39 (14.09)	42.82 (18.61)
**Height(cm)**	177.38 (7.76)	164.51 (7.22)	178.41 (8.06)	165.45 (6.58)
**Weight(kg)**	79.51 (18.57)	69.53 (17.14)	81.87 (17.98)	69.23 (17.42)
**BMI (kg/m**^**2**^**)**	25.19 (5.30)	25.67 (6.44)	25.59 (4.69)	25.28 (6.08)

Yrs = years, cm = centimeter, kg = kilograms, BMI = body mass index.

()Standard deviation.

### Prevalence and associations of overweight and obesity

Totally for this study, 224/497, 45.1% were overweight or obese (≥ 25 kg/m^2^). A number of 133/497, 26.8% were overweight and 91/497, 18.3% were obese. The prevalence of overweight and obesity was quite similar at the somatic departments (i.e. 163/356, 45.8%) and at the psychiatric departments (i.e. 61/141, 43.3%), see Table 2. In total, nearly half of the males (i.e. 115/243, 47.3%) and females (i.e. 109/254, 42.9%) were either overweight or obese. The prevalence of overweight and obesity was not statistically significant associated with gender or departments. In crude and adjusted odds ratio analyses, the odds for overweight and obesity was statistically significant increased for the age strata of 40–59 years compared to those being younger, OR being 1.76 (CI =1.0-3.0) and 1.73 (95% CI = 1.0-3.0), respectively (Table 2).

**Table 2:** **Unadjusted and adjusted odds ratio (OR) with 95% confidence interval (CI) for overweight and obesity**^**A **^**in relation to departments, gender and age strata (N = 497)**

	***N (%)***	***OR (95% CI)***	***Adjusted OR (95% CI)***
***Departments***			
Psychiatric	61 (43.3)	1.0	1.0
Somatic	163 (45.8)	1.11 (0.61, 1.33)	1.05 (0,66, 1.67)
***Gender***			
Males	115 (47.3)	1.0	1.0
Females	109 (42.9)	1.19 (0.84, 1.70)	1.16 (0,81, 1.67)
***Age strata (yrs)***			
18-39	21 (41.2)	1.0	1.0
40-59	30 (61.2)	1.76 (1.04, 2.98)	1.73 (1.01, 2.96)
60-79	52 (50.5)	1.54 (0.97, 2.45)	1.47 (0.87, 2.48)
≥80	12 (30.0)	0.83 (0.46, 1.48)	0.79 (0.42, 1.51)

^**A**^Category definitions are based on World Health Organizations (WHO’s) cutoffs. Underweight and normal weight = BMI ≤ 25 kg/m^2^, Overweight and obesity = BMI ≥ 25 kg/m^2^.

### Obesity-related conditions in the somatic sample

Since only one person (1/141, 0.7%) in the psychiatric sample was diagnosed with an obesity-related condition, analysis of obesity-related conditions in this subgroup was made impossible. The conditions reported in the somatic sample were: T2DM, hypertension, dyslipidemia, coronary artery disease, osteoarthritis, cerebral vascular accident and cholelithiasis. In total, 232 obesity-related conditions (69 main- and 170 secondary conditions) were registered in a little less than half of the somatic patients (i.e. 144/356, 40%). The clinical diagnosis of obesity was documented as a main diagnosis in only three of the 91 patients (3.3%) with a BMI of ≥ 30 kg/m^2^.

In crude and adjusted analyses, the odds for having obesity-related conditions were about doubled among both male- and female patients being overweight or obese compared to those being normal- or underweighted. The age adjusted odds for obesity-related conditions was in males and females 2.0 (95% CI = 1.11-3.74) and 2.1 (95% CI = 1.0-4.4), respectively (Table 3). Higher age increased the odds for obesity-related conditions, and especially in the oldest females (OR: 11.9, 95% CI = 2.9-48.5 in females 80 years or more).

**Table 3:** Unadjusted and adjusted odds ratio (OR) with 95% confidence interval (CI) for having obesity-related diagnoses at somatic departments by gender (N = 356)

	***Males***		***Females***	
	***Crude OR***	***Adjusted OR***	***Crude OR***	***Adjusted OR***
***BMI-categories***^**A**^				
**Under/normal**	1.0	1.0	1.0	1.0
**Over/obese**	1.84 (1.03, 3.26)	2.03 (1.11, 3.74)	1.97 (1.03, 3.77)	2.09 (0.99, 4.41)
***Age strata (yrs)***				
**18-39**	1.0	1.0	1.0	1.0
**40-59**	1.90 (0.55, 6.59)	1.62 (0.46,5.72)	0.73 (0.14, 3.61)	0.66 (0.13, 3.32)
**60-79**	3.80 (1.32, 10.97)	3.71 (1.27, 10.85)	4.10 (1.09, 15.37)	3.48 (0.91, 13.28)
**≥ 80**	3.40 (1.07, 11.01)	3.87 (1.18, 12.66)	12.00 (2.98, 48.26)	11.89 (2.91, 48.51)

^**A**^WHO’s cutoffs. *Under/normal* = BMI ≤ 25 kg/m^2^, *Over/obese* = BMI ≥ 25 kg/m^2^.

## Discussion

As the first study to detect the prevalence of overweight, obesity and related conditions in a Norwegian inpatient sample; we found overweight and obesity common as a total of 45.1% were either overweight or obese. Those aged 40–59 years had a higher risk (OR: 1.7) of being overweight and obese compared to those aged 18–39 years. Obesity-related conditions were more pronounced in 60–79 year olds and ≥ 80 year olds, for both male- (OR: 3.7 and 3.9) and female (OR: 3.5 and 11.9) patients. There was no difference between the somatic and psychiatric samples in terms of being overweight or obese. Only 3/91 patients (3.3%) with a BMI of ≥ 30 kg/m^2^ had documented a clinical diagnosis of obesity.

### Comparisons

As one could expect, the odds for being overweight or obese in the hospitalized adults were increased in those aged 40–59 years A study at an hospital in the USA have reported the highest mean BMI within the same age strata [15] and a Swedish study found obese patients to have increased risk of more acute hospital admissions and more bed days when they were in the same age strata [12].

A review has found that secular trends of the overweight and obesity-related diseases indicate the emerging disease impact of the obesity epidemic [16]. In the somatic department in our study 40.0% of the patients had one or several obesity-related conditions which is a lower proportion than reported from an hospital in the USA (75.0%) [7]. In the psychiatric department, only one patient had an obesity-related condition reported in the medical record. Studies have reported weight gain to be associated with the use of many psychotropic medications, including antidepressants, mood stabilizers and antipsychotic drugs [17], but in this study we did not have any information about medications. However, studies from the general population have shown that increased weight occurs frequently in persons with certain mental disorders, and may be particularly relevant in mood, anxiety, and personality disorders [18,19]. Also, in general population studies, a wide range of somatic diseases have been found associated with increased weight [1,4]. In the present study, we found no differences between the prevalence of overweight and obesity in somatic and psychiatric departments, which may be explained by the type of subgroups they represent of the normal population.

It may be difficult to compare studies from hospitals internationally because of differences in the organizations of the hospitals, diagnoses treated, ethnicity, age, social inequalities and disease panorama of the patients. With these restrictions in mind we would like to mention that an hospital study from USA reported an even higher mean BMI (30.8 kg/m^2^) [7] and found overweight and obesity much more prevalent [7,8] than the results from this study, which may reflect the prevalence of overweight and obesity in the USA [20]. This hospital sample, as a sub group of the general Norwegian population may reflect the rising prevalence of overweight and obesity in Norway [3,21]. However, in the present study the mean BMI at St. Olavs Hospital seemed to be lower than the mean BMI of the general population at the same time period [3]. More specifically, prevalence of overweight and obesity seemed lower and the prevalence of underweight seemed higher in the hospital compared to the general Norwegian population [3].

### Limitations and strengths

Several limitations and strengths of this study must be considered.

The present cross-sectional design gives limited ability to elucidate causal relationships between risk factors and overweight and/or obesity. Even if it is a significant difference in obesity-related conditions among overweight and obese patients compared to those with underweight and normal weight, it does not necessarily mean that it is because of overweight and obesity. It must be taken into consideration that it can be other confounding factors like smoking, physical activity, and genetic heredity that may have caused some of the conditions.

Because of the cross sectional study design we cannot say anything about age contributes to the occurrence of obesity-related conditions or if excess weight over time contributes to diagnoses. Nevertheless, we can see that obesity-related conditions were more pronounced in older patients.

Furthermore, due to the design of the study, we cannot state anything about the length of stay of hospitalization at time of data collection.

In our study, the measuring of height and weight was done with the same procedure of trained nurses. The scales were newly controlled and recalibrated so the measures of the weights were valid. The staffs could collect the data via retrospective chart reviews if this information existed (not older than two weeks). Otherwise the weight were measured rather than self-reported, which strengthens the study. Self-reported height and weight may lead to misclassification of the prevalence of obesity, as participants overestimate or underestimate height, weight or both [22].

The prevalence of overweight and obesity may look different when the different measurements of overweight and obesity are used; BMI, waist-hip-ratio, waist circumference and skin fold thickness. The WHO’s limits of BMI categories have proven to be fairly robust for classifying obesity across populations. Because it may not correspond to the same degree of fatness in different individuals it should be considered as a rough guide [1]. However, these had likely little overall impact on the analysis as the BMI was the most likely measure to use because it provides the most useful population-level measure of overweight and obesity as it is the same for both sexes and for all ages of adults.

Despite the limitations, the findings add to our understanding of the epidemiology of overweight and obesity in an adult Norwegian in-patient population. Furthermore, this study provides new data from St. Olavs Hospital, University Hospital of Trondheim, Norway.

### Future challenges

The increasing prevalence of obesity and its related conditions are likely to pose a serious challenge to the health care systems in western countries [23]. The obesity epidemic is an actual public health crisis which is global and is affecting countries all over the world both in countries with both high- and low- income [1]. This study was a beginning of the determination of the magnitude of the overweight and obesity problem in Norwegian hospitals.

According to the health burden and the fact that obesity is increasing in the Norwegian population, the results of this study indicate that obesity and related conditions may be under-documented in hospital settings. As mentioned previously, only one patient was diagnosed with T2DM in the psychiatric sample, which may indicate an under-registration in diagnoses of psychiatric patients. Also the rate of patients diagnosed with obesity may have been under-documented when only a few patients were diagnosed with obesity. Svendsen et.al found that obese patients with T2DM are seldom correctly registered with the secondary diagnosis of obesity [24]. Also in one hospital in USA they found that obesity was documented in only a few portions of the obese patients [7]. In another hospital from USA were 49% of admitted patients obese, and obesity was only documented in 19% of the admission notes [25]. Results from studies among providers suggest that providers do not document obesity because it is not considered to be an acute issue. They also elect not to address obesity because of three main reasons; they lack the time, the knowledge, and they are in believes that their efforts will be unsuccessful [25]. It is also possible that health care professionals pay more attention to the complications of obesity rather than to obesity itself.

A question that still remains unanswered is whether overweight and obese patients have longer hospital stays in Norwegian Hospitals. Future studies in Norway should try to investigate connections between overweight, obesity and duration of hospitalization. Literature from other countries has found higher proportion of hospitalizations related to obesity-associated conditions [11,12,26,27]. Additional, studies have found hospital-treatment costs higher for obese and overweight patients than normal weight patients [10,11,27,28]. It would also be interesting to get more knowledge about the use of medications, and equipment related to overweight and obese patients in Norwegian hospitals, which also is an aspect of economic impact. Assessment of the infrastructure; the use of beds and workload on patients with increased weight may be a future challenge for the hospitals. Overweight and obese patients may already be an undocumented problem affecting both hospitals and the primary health care. More research would help to address the burden of overweight and obesity in Norway. It had been additional knowledge to repeat the study in Norwegian hospitals and conduct a multicenter study in the future to see differences in time trends and longitudinal investigate the development in prevalence of overweight, obesity and related diagnoses in hospitalized patients. Regular time trend monitoring of overweight, obesity and related conditions are essential regarding treatment, management and to initiate preventive strategies.

## Conclusions

In summary, excess body weight appears to be quite common among somatic and psychiatric patients at a Norwegian Hospital. There were no differences between the somatic and psychiatric sample regarding the prevalence of overweight, obesity or related diseases. We found middle aged hospitalized people to be more associated with being overweight and obese than their younger counterparts. Regardless of age; overweight and obesity are independent associated with having obesity-related conditions in the somatic sample.

If the prevalence rates of overweight and obesity in Norway continue to increase we may see a high amount of lifestyle diseases at the hospitals in the future. This study encourages further research to increase knowledge of lengths of stay and uses of resources related to overweight and obese patients in Norwegian hospitals. How much the epidemic of overweight and obesity influences the Norwegian health care system is still unknown.

## Competing interests

The authors declare that they have competing of interest.

## Authors’ contributions

BK conceived the idea of the study in collaboration with IF and ASH. ISF drafted the manuscript and ASH supervised writing and critically reviewed the article. All authors were involved in determining the main contents of the article. All authors read and approved the final manuscript.
